# Using Dyes for Evaluating Photocatalytic Properties: A Critical Review

**DOI:** 10.3390/molecules20010088

**Published:** 2014-12-23

**Authors:** Malka Rochkind, Sagi Pasternak, Yaron Paz

**Affiliations:** Department of Chemical Engineering, Technion—Israel Institute of Technology, Haifa 3200003, Israel; E-Mails: small22@tx.technion.ac.il (M.R.); sagipas@tx.technion.ac.il (S.P.)

**Keywords:** photocatalysis, dyes, rhodamine B, sensitization

## Abstract

This brief review aims at analyzing the use of dyestuffs for evaluating the photocatalytic properties of novel photocatalysts. It is shown that the use of dyes as predictors for photocatalytic activity has its roots in the pre visible-light activity era, when the aim was to treat effluents streams containing hazardous dyes. The main conclusion of this review is that, in general, dyes are inappropriate as model compounds for the evaluation of photocatalytic activity of novel photocatalysts claimed to operate under visible light. Their main advantage, the ability to use UV-Vis spectroscopy, is severely limited by a variety of factors, most of which are related to the presence of other species. The presence of a second mechanism, sensitization, diminishes the generality required from a model contaminant used for testing a novel photocatalyst. While it is recommended not to use dyes for general testing of novel photocatalysts, it is still understandable that a model system consisting of a dye and a semiconductor can be of large importance if the degradation of a specific dye is the main aim of the research, or, alternatively, if the abilities of a specific dye to induce the degradation of a different type of contaminant are under study.

## 1. Introduction

Over the years enormous numbers of manuscripts have been published on the application of photocatalysts for water and air decontamination, as well as for maintaining clean and superhydrophilic surfaces. As part of this scientific endeavor thousands of compounds have been tested [1], demonstrating the versatility of photocatalysis and its inherent non-preferential nature, which is closely connected to the radical mechanism involved in the photocatalytic degradation process.

Among the many compounds that served, and still serve, to evaluate photocatalytic activity, are organic dyes. Dyes are usually categorized according to their chromophores. Table 1 presents the main categories of dyes and an estimation regarding the number of publications on their photocatalytic degradation under UV and under visible light. The estimation is based on the SciFinder^TM^ data source, taking the words “photocatalysis” “visible light” (or “UV light”) and “a specific name of dye (in both its formal and its commercial name)” as keywords. The table is based on data obtained for 250 different dyes. The most studied dyes are the thiazine dyes (with a dominance of methylene blue: 37% of all papers on thiazines), second to them are the xanthenes (with a dominance of rhodamine B: 30% of all manuscripts on xanthenes). Azo dyes, despite their dominance in global production (50%–70% of the market), hence their dominance in contributing to the environmental challenge, come only fourth. From the table it is evident that for all dye categories the number of manuscripts on visible light photocatalysis of dyes is larger than that of the number of manuscripts on UV light photocatalysis. The higher ratio between the number of manuscripts on visible light photocatalysis and UV light photocatalysis is found for xanthenes (2.18) and thiazines (1.80). The ratio is in particular low for azo dyes (1.56). In what follows a rationalization for this difference in the ratios is proposed.

**Table 1:** An estimation regarding the number of publications on the photocatalytic degradation of dyes under UV light and under visible light, organized according to dye categories. The estimation is based on SciFinder^TM^ data source, taking the words “photocatalysis” “visible light” (or “UV light”) and “a specific name of dye” as keywords.

Class	UV	Visible
Anthraquinones	238	390
Azo dyes	1285	2006
Natural dyes	187	303
Thiazines	7496	13471
Triarylmethanes	1439	2758
Xanthenes	5625	12244
Others	303	557

Monitoring the photocatalytic degradation of dyes as a tool for demonstrating the technological benefits of photocatalysis is quite common today. Indeed, recent years have shown a tremendous increase in the number of manuscripts describing the photocatalytic degradation of dyes. And yet, a sense of dissatisfaction from this situation appears in private communications, in scientific conferences and during conversations among peers. It is for this reason that we decided to dedicate this manuscript to analyze the sources of the use of dyes in photocatalysis and to summarize the pros and cons in taking the photocatalytic degradation of dyes as a probe for the general properties of photocatalysts.

## 2. The Mechanisms

**Figure 1:**
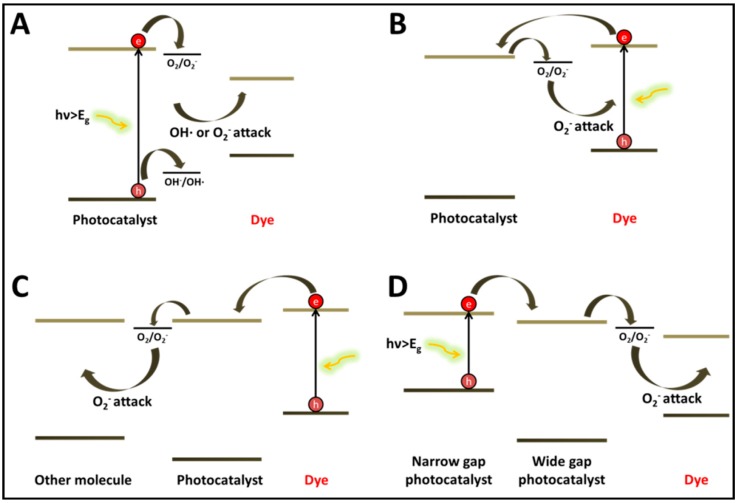
The mechanisms of light-induced degradation of dyes (**A**) photocatalysis (**B**) dye sensitization followed by dye degradation (**C**) dye sensitization followed by reduction of a second molecule; (**D**) degradation by coupled semiconductors under visible light.

The first two decades of research on the photocatalytic degradation of dyes were characterized by the dominance of un-doped titanium dioxide, a UV active photocatalyst. The general degradation scheme of dyes by the UV-active titanium dioxide (Figure 1A) consisted of photon absorption by the photocatalyst, charge separation and the generation of active species on the surface of the photocatalyst. Generally speaking, the main active species under this mechanism are OH radicals formed by oxidation of water molecules by the photogenerated holes, hence the primary attack of the dye molecules is oxidative [2,3]. Evidence for direct oxidative attack by holes was also recorded. Likewise, irreversible reductive decolarization initiated by electrons or by superoxides formed on the photocatalyst’s surface can be quite efficient, as was found for azo dyes [4,5].

Decolorization can take place also by a self-sensitization mechanism (Figure 1B). Here, the light is absorbed by the dye molecule. Charge transfer from the excited dye molecule to the conduction band of the semiconductor results in the formation of an unstable dye cation radical and in parallel an active specie on the semiconductor surface that attacks the destabilized dye molecule. One of the first demonstrations of this mechanism, published as early as 1977, described highly efficient *N*-deethylation of rhodamine B adsorbed on CdS [6]. Likewise, the fact that de-coloring kinetics of methylene blue under solar light in the presence of (undoped) TiO_2_ was faster than de-coloring kinetics under UV light was explained by this self-sesnsitization mechanism [7].

An in-depth insight into the mechanism involved in self-sensitization was presented by Liu *et al.*, who studied the photooxidation of alizarin red in TiO_2_ under visible light, combining ESR spin-trapping technique with molecular orbitals calculations [8]. It was found that the main active species was O_2_^−^ or OOH. Of large interest is their claim that the electron transferred from the dye to the semiconductor is likely to arrive from the atom having the largest electron density in the ground state. Later, this atom becomes the site where the attack by the superoxide anions radicals, formed at the surface of the semiconductor, takes place. Therefore, one may identify two major differences between the photocatalytic mechanism (Figure 1A) and the photosensitization mechanism (Figure 1B): the type of active species and the existence of a preferential location of attack in the sensitization scenario.

Of particular interest is a third photocatalytic mechanism (Figure 1C). Here, absorption takes place on the dye, as in scheme B, however the electrons transferred to the semiconductor are utilized to reduce another molecule [9]. As an example, thionine and eosineY adsorbed on TiO_2_ were able to photoinduce the degradation of phenol, chlorophenol and 1,2-dichloroethane [10,11]. A study on the degradation of aromatics, trichloroethylene and surfactants by rhodamine B and methylene blue‒adsorbed TiO_2_ suggested that the active species formed upon charge injection from the dye to the semiconductor are *O_2_^−^/*HO_2_ [12]. It should be noted that some degradation of the sensitizers may occur in parallel to the degradation of the non-absorbing contaminants. This undesired process becomes more important at low concentrations of the non-absorbing contaminants.

One of the main benefits of using a dye sensitizer attached to a semiconductor is the ability to induce chemical changes in a controlled, selective, manner. Such a control can be achieved by utilizing the fact that the location of the HOMO‒LUMO levels relative to that of the photocatalyst depends on the specific dye that is being used. For example, TiO_2_ sensitized by phthalocyanines was able to degrade, upon absorbing visible light, phenol, thiophenol, 4-chlorophenol and hydroquinone, but not oxalic acid, benzoquinone, or EDTA [13].

A narrow bandgap semiconductor coupled to a wide band gap semiconductor may serve as a sensitizer, provided that the conduction band of the wide bandgap semiconductor is more anodic than that of the sensitizer (Figure 1D). An example is the degradation of acid orange II in a coupled CdS/TiO_2_ photocatalysts under visible light [14]. A question mark might be raised justly regarding the possible role of light absorption by the dye. Comparing the kinetics to that in the presence of only one semiconductor may assist in understanding the role of the dye. For the specific system of acid orange II/CdS/TiO_2_ the slower kinetics measured in the presence of only one type of semiconductor, convinced the authors that light absorption by the CdS played the dominant role.

Our discussion of the photoinduced degradation mechanisms of dyes will not be completed without mentioning direct photolysis, *i.e.*, the degradation of the dye due to absorption of photons, without a need for a photocatalyst [15,16]. Testing of direct photolysis of dyes is performed routinely as part of the developing of a dye or a dye formulation, and assessing of product stability under weathering conditions. Likewise, photolysis tests are routinely performed and reported, as control-experiments, in almost all manuscripts dealing with photocatalysis, whether under UV light or under visible light.

Total mineralization can be achieved with most dyes [17,18]. This is not the case for dyes containing the triazine group, where the highly stable cyanuric acid is formed [19]. In azo dyes color disappearance usually reflects an attack on the azo bond (C-N=N-) [20]. This usually precedes the opening of the aromatic rings [21,22]. Therefore, aromatic amines or phenolic compounds are often observed as intermediate products. The opening of the aromatic (in other cases naphthalene rings) yields a variety of carboxylic acids, which eventually decarboxylate by the “photo-Kolbe” reaction to yield CO_2_. It should be noted that azo dyes containing a phenyl azo substitution (naphtol blue, chromotrope 2R, *etc.*) are likely to generate benzene as an end product when degraded by hydroxyl radicals [23].

It was established that dyes containing sulfur atoms are mineralized into sulfate ions [19,24]. The kinetics of sulfate formation was found to be only slightly slower than that of decolorization. Chlorinated dye molecules release chloride ions, already at the beginning of the photocatalytic process. Since chlorinated compounds cause problems in biological treatment, it was claimed that the early release of chlorides advocates for the use of a combined AOP- biological treatment, where a short photocatalytic step precedes an activated sludge treatment step [25]. Dyes containing nitrogen may release NH_4_^+^, NO_3_^−^ and even N_2_, depending on the initial oxidation state of the nitrogen atoms. Generally speaking, amino groups, consisting of nitrogen in its -3 oxidation state, produce NH_4_^+^. Once formed, the ammonium ions may slowly be photocatalytically oxidized into nitrate ions [22]. In contrast, N_2_ is the favorite end-product in the degradation of azo bonds, where each nitrogen atom is in its +1 oxidation state.

## 3. Studying Photoinduced Dye Degradation: The Motivation

### 3.1. The Early Days

Originally, the motivation to study the photocatalytic degradation of dyestuffs was explained by an actual need to treat contaminated wastewater released by the textile industry [25,26,27] claimed to be responsible for the release of as much as 15% of the total world production of dyes [28]. Other industries that release considerable amounts of dyestuff are the leather tanning industry [29], the paper industry [30], the hair-coloring industry [31] and the food industry [32]. While many of the dyes are considered toxic by themselves, the toxicity of the raw materials used for their synthesis (in particular aromatic amines) might be of larger concern [33]. Apart from toxicity, one needs to add the obvious (non)aesthetic properties of the streams; properties that are assets to photographers, journalists and politicians.

This rationalization, *i.e.*, the need to degrade large number of different contaminants paved the way for the study the photocatalytic degradation of a large number of dyes. The dominance of un-doped TiO_2_, active only under UV light in the photocatalytic arena, was clearly mapped onto the research performed on photocalytically induced dye degradation. Accordingly, almost all published data refers to scheme A, and was dominated by those dyes that pose an environmental problem. The research was mainly concentrated in monitoring the kinetics of disappearance of the primary substrate (Langmuir- Hinshelwood kinetics was mostly reported, manifesting the role of adsorption [34,35]) as well as studying the factors affecting the photocatalytic degradation rates. These included initial dye concentration (an optimal concentration was found in many cases [36,37]), solution's pH [2,38] (which affects adsorption of charged dye molecules), light intensity [39,40], addition of oxidants such as H_2_O_2_ and S_2_O_8_^2−^ [24,41], and the presence of co-existing ions [42].

The notion in the early days that the main aim in studying the photocatalytic degradation of dyes was to treat water that had been contaminated by dye-stuff and not as a means to evaluate performance of photocatalysts was echoed also by the large number of manuscripts examining the toxicity of the intermediates and that of the end-products. Methods included the monitoring of the bioluminescence of the bacteria *Vibrio fisheri* [43] or, alternatively, by following the inhibition of bacterial respiration [44,45]. Results varied according to the specific dye. For example, Reactive Black 5 emitted toxic by-products [46], whereas the intermediate products of the triazine dyes MX-5B and K-2G were found to be non-toxic [19,39].

The introduction of self-cleaning surfaces, *i.e.*, self-cleaning glass [47,48], self-cleaning cementitious materials [49,50], and self- cleaning fabrics [51] marked a change in the role that dyes played in pohotocatalysis. For these applications, the dyes no longer served as actual contaminants that had to be degraded in order to protect from their own toxicity, but rather as a tool for demonstrating the potential of photocatalysis as a means to protect aesthetic assets. Indeed, the Japanese industrial standard JIS-R-1703-2:2007 utilizes methylene blue for evaluation of self- cleaning surfaces [52]. Choosing dyes for such demonstrations was a natural choice. After all, the main purpose was to preserve the original color and to prevent color change due to weathering. Intermediate products, mineralization, adsorption were no longer of importance. The only important characteristic was now the kinetics of de-coloring. This focus change was manifested by a change in the scientific tools monitoring the photocatalytic activity. Infrared spectroscopy, HPLC, GCMS and NMR became almost obsolete for these applications. Instead, visible light spectrophotometery, a non-expensive, easy-to-use technique became the technique of choice. A direct step further was the use of human eyes as indicators for decoloration of dyes, thus presenting photocatalysis to laymen, and, no less important, to policy makers. Often, the surfaces of large objects like walls or pavements were colored with dyes and exposed selectively, thus stressing the photocatalytic effect to the human eye, which, by nature, is very sensitive to slight differences in color and tint at domain boundaries.

### 3.2. The Second Phase- Developing New Materials Operating under Visible Light

The introduction of photocatalysts that respond to visible light marked a significant change in the study of photocatalytic degradation of dyes. Originally, visible light response was achieved by manipulating the activity of titanium dioxide either by doping with non-metallic elements (in particular nitrogen [53,54], carbon [55], sulfur [56,57], fluorine [58], and their mixtures [59]), by doping with transition metals [60], and by coupling the photocatalyst with other semiconductors [61]. In parallel to the manipulation of titanium dioxide a zeal for developing new photocatalysts having narrow bandgaps has been noticed. Apparently, this trend occurred (actually still occurs) in parallel with the developing of the Dye Sensitized Solar Cells (DSSC) and with the renewed interest in hydrogen production by photoinduced water splitting. Many of the proposed materials are ternary and quaternary oxides of bismuth [62,63,64].

A very common means to evaluate the properties of the new photocatalysts is reflection spectroscopy, from which apparent bandgap values can be calculated based on Tauc plots [65]. Without getting into whether doping or any other photocatalyst’s manipulation is manifested by band narrowing or by introduction of energy levels within the bandgap, it is quite obvious that absorbing visible light does not necessarily imply photocatalytic response to that light. This is partly because of high recombination rates and partly because of energy mismatch that thermodynamically prevents the formation of critical species such as hydroxyl radicals or superoxides. For this reason there is a constant need for generic method to evaluate the visible light activity of the new photocatalysts.

In light of this need, the photoinduced degradation of dyes (and in particular rhodamine B, methylene blue) became in recent years the preferred way to evaluate the photoactivity of the novel, apparently visible-light-active materials. In other words, the main reason for studying the photocatalytic degradation of dyes was no longer the need to treat contaminated water but rather their potential use as a probe for the activity of the novel photocatalysts.

**Figure 2:**
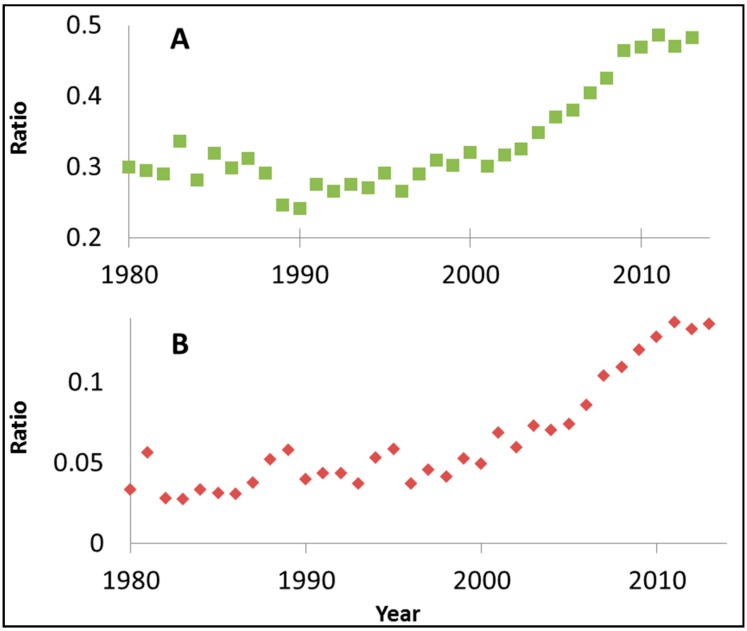
(**A**) The ratio between the number of manuscripts retrieved by SciFinder^TM^ database upon using the keywords “Visible light” and “Photocatalysis” and the number of manuscripts retrieved by using only “Photocatalysis” as a keyword; (**B**) The ratio between the number of manuscripts retrieved by the SciFinder^TM^ database upon using the keywords “dye” and “Photocatalysis” and the number of manuscripts retrieved by using only “Photocatalysis” as a keyword.

That the popularity of using dyes has increased since the introduction of photocatalysts operating in the visible part of the spectrum is demonstrated in Figure 2. Figure 2A presents, on an annual basis, the ratio between the number of manuscripts retrieved by SciFinder^TM^ database upon using the keywords “Visible light” and “Photocatalysis” and the number of manuscripts retrieved by using only “Photocatalysis” as a keyword. An abrupt increase in that ratio is observed around years 2002–2004, marking the change in focus from photocatalysts operating under UV light (mostly undoped titania) to photocatalysts claimed to operate under visible light. Likewise, Figure 2B depicts the ratio between the number of manuscripts retrieved by the SciFinder^TM^ database upon using the keywords “dye” and “Photocatalysis” and the number of manuscripts retrieved by using only “Photocatalysis” as a keyword. A striking similarity between the two graphs is noticed, supporting the linkage made by us between the developing of photocatalysts operating under visible light and the use of dyes as model contaminants.

Using any model contaminant as a probe for effectiveness of a new photocatalyst requires that the degradation kinetics, whatever they are, will be, as much as possible, generic, in a sense that comparative studies performed on a set of photocatalysts will have a relevance (at least partially) for the degradation of other contaminants. In practice, most manuscripts do not report that the degradation they measured under visible light could be due to photosensitization rather than photocatalysis. In many cases, even when sensitization is mentioned as the governing mechanism, the (ir)relevance for other contaminants is only rarely mentioned.

One of the first studies pointing out the inadequacy of dyes as a probe molecule for semiconductor photocatalysis (methylene blue in this case) was presented by Yan *et al.* [66]. Here, the action spectra of *S*-doped TiO_2_ measured during the degradation of methylene blue revealed activity at 580–650 nm (in correlation with the absorption spectrum of MB), whereas no activity at this range of the spectrum was observed towards acetic acid. The same group repeated its concern about the use of dyes for evaluating activity also at a later manuscript [67]. Recently, a clear recommendation not to use dye tests for activity assessment of visible light photocatalysts was presented, based on experiments with six visible- light photocatalysts degrading five organic dyes [68].

Basic ethics of integrity require that comparative studies, which are most likely relevant to one contaminant only, will be reported as such, and will not be publicized in a generalized manner that misleads the readers. It is quite unfortunate that, generally speaking, these basic ethics are not strictly obeyed. Was this behavior a matter of misinterpretation of data? Of innocent misunderstanding of the role that dye sensitization played in the obtained kinetics? Was it due to a sloppy scientific community that did not emphasize enough the difference between sensitization and “true” photocatalysis? This is not for us to judge. What we can do (and we do it hereby) is to analyze the factors that render dyes inappropriate for serving as generalized indicators for photocatalytic activity, and to discuss possible remedies for this situation.

This manuscript is basically a mini-review manuscript. As such, it relies on published articles and on work made by large number of research groups. While relying on the work of others, we thought it would be beneficial to perform a set of experiments that would demonstrate our claims and would augment our conclusions. In doing so we aimed at providing common grounds to the presented claims, knowing that while there are many reviews on the photocatalytic degradation of dyes, the published reviews basically summarize the kinetic and mechanistic results and are quite silent in giving a critical view on the fundamentals of studying the photocatalytic degradation of dyes.

## 4. Rhodamine B and BiOCl

**Figure 3:**
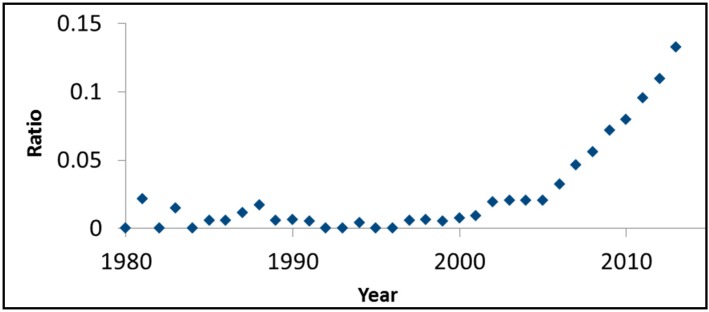
The ratio between the number of SciFinder™ hits for “rhodamine B” + “visible light” + “photocatalysis” and the number of hits for “visible light” + “photocatalysis”.

The photocatalytic degradation of rhodamine B (*N,N,N',N'*-tetraethylrodamine) under both UV light and visible light in the presence of bismuth oxychloride (BiOCl) was used as a model system. Rhodamine was chosen based on its popularity among research groups studying visible light activity. Figure 3 presents, on an annual basis, the ratio between the number of SciFinder™ hits for rhodamine B and visible light photocatalysis and the number of hits for visible light and photocatalysis. Although this ratio should not be regarded as more than a rough estimation, the monotonic increase as a function of time and the high values obtained (0.13 in 2013, for example), are indicative of the important role of this dye in evaluating the photoactivity of the so-called visible light photocatalysts.

Rhodamine B belongs to the oxygen-containing heterocyclic xanthene dyes family. It is neither degraded in the dark in the presence of a photocatalyst nor under illumination in the absence of a photocatalyst. Under visible light, and in the presence of an appropriate semiconductor, rhodamine B degrades via an efficient N-deethylation sensitization mechanism. The process is initiated by sensitization of the dye, charge transport to the semiconductor and formation of active superoxide ions by reduction of oxygen pre-adsorbed on the semiconductor. Consequently, in this scenario the degradation of rhodamine B goes through the formation of triethylrhodamine, diethylrhodamine, ethylrhodamine and rhodamine having different λ_max_ at 555, 539, 522, 510, 498 nm, respectively and different relative extinction coefficients of 1:0.48:6.3:5.3:0.73, respectively [6]. At a later stage the nascent rhodamine is mineralized. Some photocatalysts, which absorb visible light, such as Bi_2_O_3_ (Eg = 2.85 eV) and BiVO_4_ (Eg = 2.44 eV) tend to degrade RhB under visible light, not by a sensitization mechanism but by a photocatalytic mechanism which is manifested by a direct attack on the chromophore. This behavior was explained by the weak interaction between these photocatalysts and the dye, which prevents charge transport from the dye to the photocatalyst [69].

The photocatalyst used by us in the present work was BiOCl, consisting of tetragonal Bi_2_O_2_ slabs, “sandwiched” between two chlorine ions slabs to form a Bi_2_O_2_Cl_2_ layer along the c-axis. This structure forms internal electric fields between the Bi_2_O_2_ positive slabs and the halide anionic slabs that promote the separation of photogenerated electron-hole pairs thus improving the photocatalytic activity of the catalyst [70,71,72]. Unlike other bismuth oxyhalides the bandgap of BiOCl is large (3.2–3.5 eV) [73], thus it does not absorb in the visible part of the spectrum. For this reason any degradation induced by visible light can be regarded as originating from sesnsitization, whereas, by virtue of the small absorption cross section of the dye at 365 nm, degradation by UV light is likely to occur predominantly due to photocatalysis. Unlike TiO_2_, which hardly physisorbs the rhodamine dye, BiOCl is known to chemically adsorb the dye [74], facilitating easy charge transport from the dye to the semiconductor.

### 4.1. Preparation of BiOCl

BiOCl was prepared by co-precipitation. 1.3 gram of BiCl_3_ were dissolved in 20.5 mL of 1.23 M HCl to obtain bismuth solution concentration of 0.23 M. 18 ml of NaOH 2 M solution were added to the bismuth solution to obtain a pH of 11.5. The solution was then stirred at room temperature for 24 h. The particles were then collected by vacuum filtration, washed with water and dried in air. The material was characterized by XRD and SEM.

### 4.2. Photocatalytic Tests

BiOCl was used to study the photocatalytic and photosensitized degradation kinetics of Rhodamine B (RhB) in aqueous solutions. In a typical experiment, 30 mg of the photocatalyst were dispersed in 50 mL solution containing 1.25 × 10^−5^ M (in other cases 3.1 × 10^−5^ M) of RhB, in a reaction vessel under continuous stirring. Prior to exposure, the system was left in the dark for 90 min to obtain an adsorption/desorption equilibrium. Upon reaching equilibrium the solution was exposed to light at a specific wavelength in the range of 365–515 nm. A series of Light Emitting Diodes (LED-R Ltd., Ben-Shemen, Israel) having a typical 15 nm FWHM were used. Equal photon flux (3.17 × 10^15^ photons/(s·cm^2^)), rather than equal energy flux, was kept in all experiments. At given time intervals, 0.67 mL aliquots were sampled, centrifuged to remove the particles and measured by UV-vis spectroscopy (Lambda 40, Perkin-Elmer, Waltham, MA, USA).

## 5. Using Dyes for Evaluating Photocatalytic Properties

### 5.1. Monitoring the Degradation Kinetics

There is a general acceptance of the notion that the most important characteristic of a photocatalyst is its ability to degrade molecules of interest as fast as possible, at a given number of impinging photons and with a given amount of photocatalyst. This, almost automatically, raises a question regarding the definition of “degrading”. Is it the kinetics of decoloring or, alternatively, the kinetics of mineralization?

As mentioned above, in the early days of photocatalytic dye degradation the main aim was the remediation of wastewater containing the specific dyes that were tested. Consequently, both de-coloring kinetics, types and formation/degradation kinetics of intermediate products, and mineralization rates were studied. Apparently, altering the focus into using the degradation of dyes as an indicator for the photoactivity of new photocatalysts operating under visible light reduced the importance of studying the fate of intermediates. Indeed, if one surveys the literature reporting on the developing of novel photocatalysts one finds mainly decoloring experiments and hardly any data on intermediates and their toxicity as well as on the rates of mineralization. Such an approach might be problematic, as explained below.

Dye molecules are usually comprised of relatively large number of functional groups. This may lead to numerous types of mechanisms, each releasing different intermediate products. Therefore, in comparing two different photocatalysts the rates of decolorization do not necessarily correlate with mineralization rates. Usually, the absorption spectrum of these intermediates partially overlaps with that of the dye; the extent of interference depends on the type of the intermediates, or, in other words, on the degradation mechanism. Since the mechanism is photocatalyst-dependent, estimating the rates of decoloration by monitoring a specific absorption peak inherently gives erroneous results that are influenced by the type of photocatalyst in use. This statement was clearly demonstrated in the study of visible-light photocatalytic degradation of methyl orange on carbon-doped TiO_2_ and on Pt/WO_3_ [68]. Here, degradation on carbon-doped TiO_2_ that proceeded via demethylation yielded intermediates that absorbed light at higher energies (hence caused the blue-shifting of the peak [75]), yet partially interfered with the main peak in the absorption spectrum of the dye (505 nm). In contrast, degradation on the Pt/WO_3_ photocatalyst did not yield any intermediate products that absorbed light in the visible part of the spectrum.

**Figure 4:**
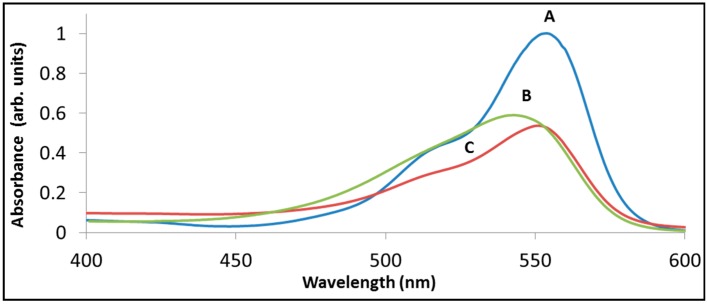
The degradation of rhodamine B with BiOCl (B) and with La_2_BiNbO_7_ (C) under visible light. Trace (A) presents the spectrum of RhB prior to degradation, peaked at 554 nm. Traces (B) and (C) present the spectrum at a point where 48% of the initial absorbance at at 554 nm has disappeared.

Figure 4 demonstrates the above statements by presenting the spectrum of rhodamine B following sensitized degradation under visible light by BiOCl (trace B) as well as by La_2_BiNbO_7_ (trace C). While both traces have the same absorbance at 554 nm (52% of the original) the two spectra are quite different. In fact, at this stage the maximum absorbance with La_2_BiNbO_7_ remained at 554 nm, whereas with BiOCl the maximum was located at 544 nm.

Hence, comparing between two photocatalysts by monitoring changes in the height of their main absorption peak might lead to flawed conclusions. The fact that, in general, the extinction coefficients of the partially overlapping intermediate products differ from that of the dye adds to the complexity of obtaining reliable kinetic data based on single wavelength Beer’s law.

Plotting the absorbance at the center of the absorption peak of a dye is no doubt the easiest way to describe the decoloration kinetics. Such a representation reveals an exponential decay curve, in almost all cases, which enables to describe the kinetics by a single number, *i.e.*, by a first order rate constant. While we have our reservations as for the validity of these values in describing the so-called “activity” of the semiconductor (see below), we are not able to suggest a better way of representation. Hence, in what follows, we will not avoid using “k”, the apparent first order rate constant of decoloration as a representative parameter of the process.

As discussed above, decolorization can be achieved by a reductive mechanism. The reduced form is then further degraded (usually by an oxidative pathway) to form the end-products. In certain cases, for example in the degradation of methylene blue, and under appropriate conditions (absence of light, high pH, high concentration of dissolved oxygen) the photoreduced specie might be re-oxidized back to colored methylene blue [76,77]. This observed (at least partial) reversibility, and in particular its dependence on the experimental conditions, marks methylene blue (and any other dye which behaves similarly) as inadequate for testing photocatalytic activity.

### 5.2. Sensitization Versus Photocatalysis

In light of the possible co-existing of a photocatalytic mechanism and a sensitization mechanism in the degradation of dyes it is required to analyze the relative contribution of dye sensitization *versus* that of “true” photocatalysis. Comparing the absorption spectrum of the photocatalyst, the absorption spectrum of the dye and the action spectra during the degradation of the dye is often suggested as a tool for obtaining such analysis. The expectation is that sensitization occurs at energies that are not absorbed by the photocatalyst and that the sensitization activity correlates with the absorption spectrum of the dye.

The fact that sensitization involves charge transport from the dye molecule to the semiconductor implies that adsorption should play a critical role in the process. The type of interaction (physisorption *versus* chemisorption) and (in the case of chemisorption) even the specific interaction between the adsorbate and adsorbent may significantly affect the efficiency of the process [78].

Charge transport from the photosensitized dye to the photocatalyst (and in the other direction as well) depends on the strength of interaction between the dye and the surface of the photocatalyst [79]. For this reason, the kinetic barrier for one electron transfer from an eosin Y dye to the conduction band of titanium dioxide, is lower than that for triethanolamine (TEOA), although the latter is a stronger reductant (under light and in the absence of a semiconductor triethanolamine can reduce free eosin Y) [12,80]. As a consequence, visible light illumination of TiO_2_ in the presence of both eosin Y and TEOA degrades the triethanolamine by a mechanism that involves electron transport from the TEOA to the cation form of the adsorbed dye.

Therefore, surface treatments that promote this interaction (for example pre-treatment of TiO_2_ with water [81], or altering the bridging group by fluorinating the surface of titanium dioxide [82]) are likely to improve sensitization-induced degradation. The strength of the interaction is likely to vary from one photocatalyst to the other. Hence, the relative contribution of sensitization by a specific dye might depend on the specific photocatalyst. Indeed, the higher rates of degradation of rhodamine B with BiOCl co-doped with niobium and iron compared with the rates with pristine BiOCl were explained by specific interaction between the dye molecules and the co-doped photocatalyst [64].

It could have been expected that sensitization would be manifested (at illumination energies above some threshold) by a correlation between the absorption spectrum of the dye and its action spectra (activity *versus* irradiated wavelength under a constant photon flux). This expectation is based on the pre-assumption that the quantum yield of the absorbed photons is independent of wavelength, as a result of fast intramolecular de-excitation to the bottom of the excited state band, *i.e.*, absence of hot-electrons effects. In practice, the correlation between the action spectrum and the absorption spectrum of the dye can be quite weak. For example, methylene blue has a very wide absorption peak. The extinction coefficient rises monotonically until it peaks at 660 nm. Yet, the degradation rate obeyed the following order: 570 = 640 = 670 < 540 < 605 < 555 < 585 < 525 < 620 < 655 nm [66].

**Figure 5:**
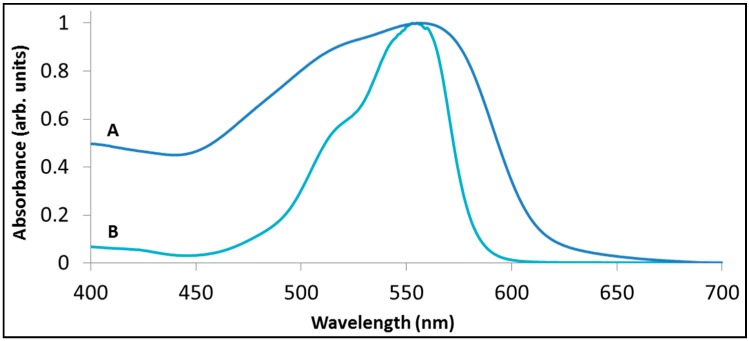
The absorption spectrum of rhodamine B (A) adsorbed on BiOCl (B) in aqueous solution.

One obvious explanation for the weak correlation between the absorption spectrum of the dye and its action spectrum stems from the fact that the parameter usually (but not always [66]) taken in the literature for this correlation is the absorption spectrum of the dye in solution instead of the absorption spectrum of the adsorbed dye. Figure 5 presents the absorption spectrum of an aqueous solution (15 mg/L) containing rhodamine B, together with the absorption spectrum of rhodamine B pre-adsorbed from the same concentration solution onto BiOCl. The latter spectrum was inferred from reflection measurements. For clarity both spectra were normalized according to their maximal absorption. It is evident that the two spectra differ in the width of their absorption peak (the adsorbed dye had a much broader peak), and, less significantly, in the position of their absorption peak (556 nm *versus* 554 nm for the adsorbed and the non-adsorbed rhodamine, respectively).

**Figure 6:**
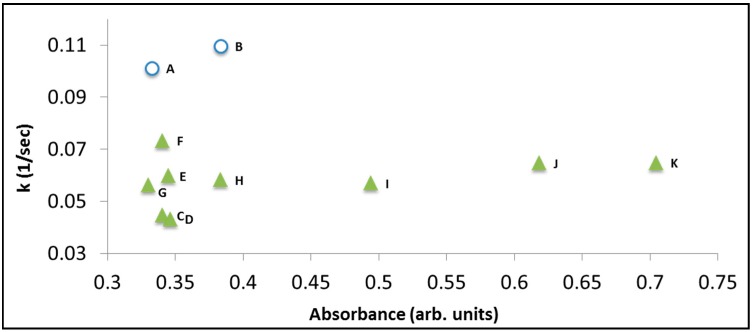
The decolorization rate constant *versus* relative absorption of RhB on the surface of BiOCl. Each point represents exposure to light of different wavelength (but same photon flux). The nominal concentration of the dye was 6 mg/L. (A) 365 nm; (B) 375 nm; (C) 395 nm; (D) 405 nm; (E) 410 nm; (F) 420 nm; (G) 435 nm; (H) 470 nm; (I) 490 nm; (J) 505 nm; (K) 515 nm.

It is noteworthy however that using the spectrum of the adsorbed dye as a source for correlation with the action spectrum still does not guarantee a high fidelity correlation. Figure 6 presents the decolorization rate constants obtained from a series of photoinduced degradation measurements of RhB (concentration: 6 mg/L) in the presence of BiOCl. Each data point represents exposure to light having a specific wavelength. The two data points at k = 0.1 sec^−1^ and k = 0.11 sec^−1^ represent exposure to photons in the UV range (365 nm and 375 nm, respectively) where the degradation mechanism is not photosensitization but photocatalysis, hence is the higher rate constant. A very weak correlation (if at all) between the absorption spectrum at the surface and the rate constant can be observed. This weak correlation disappeared upon degrading solutions with higher (15 mg/L) concentration (not shown here).

### 5.3. Effect of Dye Concentration

Another deleterious effect of utilizing dye molecules for testing visible-light active photocatalysts is the dependence of the measured kinetics on the concentration of the dye. As mentioned above, working with UV-active photocatalysts to degrade molecules that do not absorb UV light, gives, for most cases, a Langmuir- Hinshelwood type of kinetics, where the rate increases upon increasing the concentration of the contaminant until rate-saturation is achieved. In that case, the apparent kinetics reflect the number of molecules that are adsorbed on the surface of the photocatalyst. The situation is totally different in the case of a system comprising of a visible—light absorbing photocatalyst and a visible light absorbing contaminant (dye). Here, increasing the concentration of the dye might reduce the rate of degradation, due to increased absorption of light by free dye molecules in the solution [25,83]. Along this line, the higher the concentration of the dye is, the weaker is the correlation between the action spectrum and the absorption spectrum of the dye.

High concentration might also cause aggregation and even surface dimerization. In certain cases, dimerization might have a deleterious effect on the degradation rates, as was observed in the case of phthalocyanines, where the dimers are considered to be photochemically inactive [13], or in the case of indigo carmine on N-doped TiO_2_. In other cases (acid orange 7 on N-doped TiO_2_) dimerization increases the decoloration rate [68].

**Figure 7:**
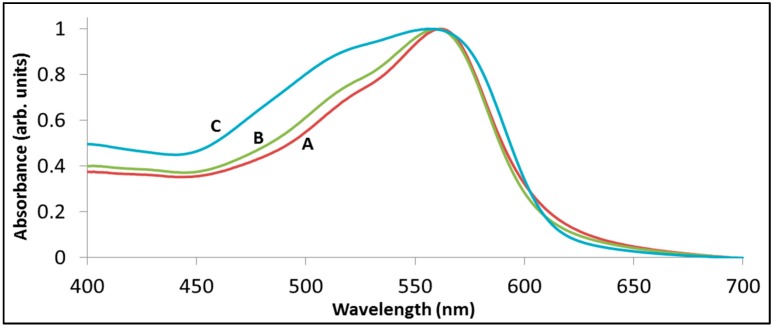
Changes in the absorption spectrum of rhodamine B pre-adsorbed on BiOCl from aqueous solutions containing various dye concentrations. For clarity, all graphs were normalized. (A) 0.5 mg/L; (B) 1.5 mg/L; (C) 15 mg/L.

Aggregation may affect the apparent decoloration rates also indirectly. Figure 7 presents the absorption spectra of rhodamine B adsorbed on BiOCl from solutions having different concentrations. A broadening of the absorption peak of the adsorbed dye to the blue upon increasing the concentration of the dye in the solution is clearly observed. Blue shifting in the absorption spectrum upon surface aggregation was reported also for Methylene Blue [52]). In that case, the higher the concentration of the dye during adsorption was, the more blue-shifting was observed. This means that the apparent degradation kinetics, often deduced based on single wavelength absorbance, are erroneous. Moreover, since the adsorpticity of a specific dye depends on the characteristics of the photocatalyst, it is obvious that the extent of concentration-dependence effects become photocatalyst-specific.

As mentioned above, more and more researchers tend to criticize reports on degradation of dyes under visible light in the presence of a visible light absorbing semiconductors claiming that they do not reflect “true” photocatalysis but sensitization. A common counter-argument is based on Total Organic Carbon (TOC) measurements showing that the degradation proceeds all the way to mineralization. It is often claimed that observing mineralization can be regarded as an evidence that the semiconductor handles not only dyes, but also the intermediate products, including intermediate products that do not absorb visible light, hence cannot induce visible-light sensitization. We believe that this argumentation does not stand on solid grounds, for two reasons. One reason is that most intermediate products are destabilized chemical species hence continue to degrade quite easily. The second reason has to do with the ability of dye molecules that are attached to the semiconductor to photoinduce degradation of other molecules (Figure 1C). Thus, observing mineralization of intermediates does not necessarily imply that these intermediates may be degraded without the presence of sensitizing molecules.

### 5.4. Monitoring Intermediate Products

As mentioned before, organic dye molecules are characterized by the presence of a chromophore having several delocalized functional groups and side-functional groups that are instrumental in defining the energetic difference between the LUMO and the HOMO orbitals. As a consequence, light-induced degradation of the dyes is often characterized by large number of intermediate steps, on way to complete mineralization. The outcome is a gradual change in the spectrum of the solution that is characterized by a decrease in the absorption at the summit of the absorption peak of the dye and, in parallel, the appearance of a broad absorption envelope, representing the intermediates. Therefore, the kinetics of degradation can be defined by several ways: (1) following changes in the absorption peak of the dye (2) by following changes in the location of the superimposed peak (3) by following changes in the amount of intermediates. Calculating the amount and type of intermediates without separating the species is very cumbersome due to a strong partial overlap between the spectra of the intermediates and that of the dye. This problem is aggravated if the extinction coefficient of the species is different.

**Figure 8:**
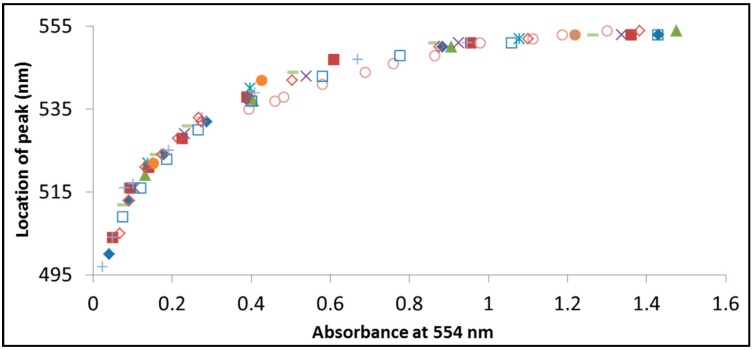
The location of the superimposed peak of rhodamine B and its intermediates during photodegradation by BiOCl *versus* the absorbption at 554 nm, which is the location of the RhB peak. Each symbol in the figure represents exposure at a different wavelength: 365 nm (filled diamonds), 375 nm (filled squares), 395 nm (filled triangles), 405 nm (× symbols), 410 nm (asterisks), 420 nm (filled circles), 435 nm (+ symbols), 470 nm (empty circles), 490 nm (− symbols), 505 nm (empty squares), 515 nm (empty diamonds).

Figure 8 presents the location of the superimposed peak of rhodamine B and its intermediates during its photodegradation by BiOCl *versus* the absorbption at 554 nm, which is the wavelength of maximum absorption for pristine RhB. Each symbol in the figure represents exposure at a different wavelength. That way, the reaction progresses from right to left. This kind of representation of the degradation progression enables to compare between the two measurable parameters in the spectrum that are changed during degradation. The figure clearly shows high level of correlation between the location of the superimposed peak and the decrease in the absorption at 554 nm, suggesting, without the need of elaborate separation equipment, that the distribution of intermediates, at a specific percentage of decolorization of the dye is the same, regardless of the wavelength of exposure.

### 5.5. Effect of Light Intensity

**Figure 9:**
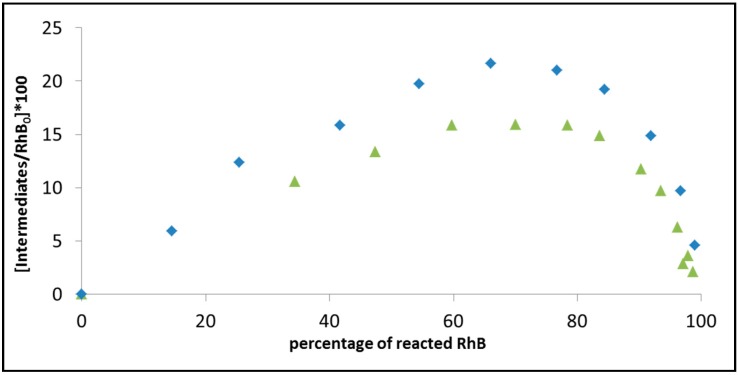
The progression of the photoinduced degradation of rhodamine B on BiOCl under 515 nm light, at two light intensities: 0.3 mW/cm^2^ (diamonds) and 1.19 mW/cm^2^ (triangles). The figure presents the calculated amount of intermediates *versus* the decrease in absorption at 554 nm. The initial concentration of RhB in both cases was 6 mg/L.

Another way to describe the progression of degradation is by plotting the calculated (actually estimated) amount of intermediates *versus* the decrease in the concentration of the dye. Figure 9 demonstrates this attitude by comparing the progression in the degradation of rhodamine B on BiOCl under 515 nm light, at two light intensities: 0.3 mW/cm^2^ and 1.19 mW/cm^2^. As in Figure 8, the intact RhB concentration was calculated based on the absorption at 554 nm. The amount of intermediates was estimated by taking the absorption at 530 nm and subtracting the contribution to the 530 nm signal from non-degraded RhB, based on the 530 nm/554 nm absorption ratio in the RhB solution prior to exposure. Care was made to take into account, while calculating the amounts of RhB and the intermediates, the difference in the extinction coefficients. According to the figure, increasing the light intensity reduces the maximal concentration of intermediates. Since the rate constant for *N*-deethylation (producing the intermediates) also increases with intensity, the lower maximum should be explained by a faster degradation of the *N*-deethylated species upon increasing light intensity that more than compensates for the increase in the rate of *N*-deethylation. It is possible that this increase in the degradation rate of the intermediates is a consequence of a sensitization process by the intermediates. Therefore, it can be concluded that secondary degradation processes might mask the primary process, thus limit the ability to deduce conclusion on the activity by following the kinetics of disappearance of a single dye. One way to detour the problem is to irradiate at a wavelength that is absorbed only by the dye, however, this solution is cumbersome.

**Figure 10:**
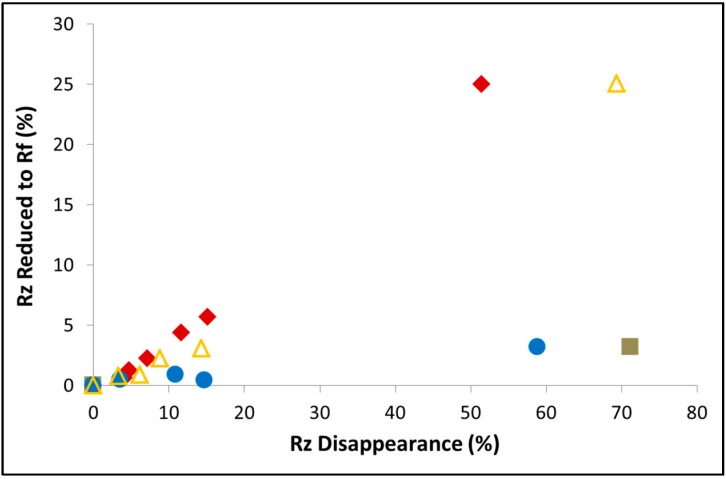
The disappearance of resazurin and the production of resorufin during photoinduced degradation of the dye with BiYWO6 prepared by various techniques: Hydrothermally prepared sample (filled squares), hydrothermally prepared sample calcined at 800 °C (filled circles), sol-gel sample calcined at 600 °C (filled rhombuses) and at 800 °C (empty triangles).

The sensitivity of the degradation mechanism in dyes to the experimental conditions does not stop at concentration, irradiance, or wavelength. In fact, the same photocatalyst can yield different mechanisms, if prepared in a different manner. The dye resazurin (Rz) is known to photocatalytically reduced to resorufin (Rf), which further degrades to non-absorbing species. Figure 10 presents the progression in the degradation of Rz upon exposure to 420 nm light (3 mW/cm^2^) in the presence of BiYWO_6_ prepared by a hydrothermal method and by a sol-gel method.

In the figure, the percentage of Rz disappearance is plotted against the percentage of formed Rf. A distinct difference in the amount of formed Rf at a given Rz disappearance is portrayed between the sol-gel-prepared BiYWO_6_ and a hydrothermally-prepared BiYWO_6_, manifested by a higher concentration of the intermediate Rf with the sol-gel prepared photocatalyst. Since the kinetics of Rz disappearance were similar for the two photocatalysts, the above difference has to be due to slower degradation kinetics of Rf with the sol-gel photocatalyst.

## 6. Conclusions

This brief review aims at analyzing the use of dye stuff for evaluating the photocatalytic properties of novel photocatalysts. As shown above, this use of dyes as predictors for photocatalytic activity has its roots in the pre-visible light activity era, when the aim was to treat effluents streams containing hazardous dyes. While dyes may be appropriate (with some limitations) for evaluating photocatalytic processes that take place under UV light and with UV-active photocatalysts, they are definitively problematic under visible light and with visible light-active photocatalysts, as discussed above.

The main conclusion of this review is that dyes in general are inappropriate as model systems for the evaluation of photocatalytic activity of novel photocatalysts claimed to operate under visible light. Their main advantage, the ability to use UV-vis spectroscopy, a non-expensive, easy to use technique, to measure the kinetics of their photocatalytic degradation, is severely limited by a variety of factors, most of which related to the presence of other species (intermediate products in the solution, dye aggregates on the surface, *etc.*). Exposure to visible light carries with it the most problematic aspect of using dyes, the presence of a second mechanism, sensitization, that diminishes the generality required from a model contaminant testing a novel photocatalyst. This conclusion is further supported by the low purity of commercially available dyes (70%–90%), that inhibits the obtaining of reliable data, and, more important, prevents making comparisons between results measured by different groups [68].

The observation that dye tests are highly specific to a dye/photocatalyst combination led to the recommendation to rely on a set of different dyes rather than on a single dye for evaluating the activity of a specific photocatalyst [68]. We believe this is definitively a step in the right direction from the current situation where novel photocatalysts are tested by their ability to degrade a single dye. The recommendation to use multitude of dyes is connected with the pH dependence of the adsorption of the dyes, however such dependence is not specific for dyes.

While it is recommended not to use dyes for general testing of novel photocatalysts, it is still understandable that a model system consisting of a dye and a semiconductor can be of large importance if the degradation of a specific dye is the main aim of the research, or, alternatively, if the abilities of a specific dye to induce the degradation of a different type of contaminant are under study. In both cases, scientific integrity demands that the relevant objectives of using dyes will be clarified by the reporting researchers. Furthermore, that sensitization will be reported as such, and that there will be no broad claims giving the readers false impression of a “true” visible light activity, when such activity does not exist. Above all, the paper calls for looking at a system containing both a specific dye and a semiconductor as a one integral system rather than a photocatalyst operating on a dye.

## References

[1] Blake D.M. (1994). Bibliography of Work on the Heterogeneous Photocatalytic Removal of Hazardous Compounds from Water and Air.

[2] Hustert K., Zepp R.G. (1992). Photocatalytic Degradation of Selected Azo Dyes. Chemosphere.

[3] Ojani R., Raoof J., Zarei E. (2012). Electrochemical Monitoring of Photoelectrocatalytic Degradation of Rhodamine B using TiO_2_ Thin Film Modified Graphite Electrode. J. Solid State Electrochem..

[4] Vinodgopal K., Bedja I., Hotchandani S., Kamat P.V. (1994). A Photocatalytic Approach for the Reductive Decolorization of Textile Azo Dyes in Colloidal Semiconductor Suspensions. Langmuir.

[5] Vinodgopal K., Kamat P.V. (1994). Photochemistry of Textile Azo Dyes. Spectral Characterization of Excited State, Reduced and Oxidized Forms of Acid Orange 7. J. Photochem. Photobiol. A.

[6] Watanabe T., Takizawa T., Honda K. (1977). Photocatalysis through Excitation of Adsorbates. 1. Highly Efficient N-Deethylation of Rhodamine B Adsorbed to Cadmium Sulfide. J. Phys. Chem..

[7] Kuo W., Ho P. (2001). Solar Photocatalytic Decolorization of Methylene Blue in Water. Chemosphere.

[8] Liu G., Li X., Zhao J., Horikoshi S., Hidaka H. (2000). Photooxidation Mechanism of Dye Alizarin Red in TiO_2_ Dispersions under Visible Illumination: An Experimental and Theoretical Examination. J. Mol. Catal. A.

[9] Taqui Khan M., Chatterjee D., Bala M. (1992). Photocatalytic Reduction of N_2_ to NH_3_ Sensitized by the [Ru III-Ethylenediaminetetraacetate-2,2'-Bipyridyl]−Complex in a Pt-TiO_2_ Semiconductor Particulate System. J. Photochem. Photobiol. A..

[10] Chatterjee D., Mahata A. (2001). Photoassisted Detoxification of Organic Pollutants on the Surface Modified TiO_2_ Semiconductor Particulate System. Catal. Commun..

[11] Chatterjee D., Mahata A. (2001). Demineralization of Organic Pollutants on the Dye Modified TiO_2_ Semiconductor Particulate System using Visible Light. Appl. Catal. B.

[12] Chatterjee D., Mahata A. (2002). Visible Light Induced Photodegradation of Organic Pollutants on Dye Adsorbed TiO_2_ Surface. J. Photochem. Photobiol. A.

[13] San Romaen E., Navío J., Litter M. (1998). Photocatalysis with Fe/TiO_2_ Semiconductors and TiO_2_ Sensitized by Phthalocyanines. J. Adv. Oxid. Technol..

[14] Bessekhouad Y., Chaoui N., Trzpit M., Ghazzal N., Robert D., Weber J. (2006). UV–vis Versus Visible Degradation of Acid Orange II in a Coupled CdS/TiO_2_ Semiconductors Suspension. J. Photochem. Photobiol. A..

[15] Bandara J., Kiwi J. (1999). Fast Kinetic Spectroscopy, Decoloration and Production of H2O2 Induced by Visible Light in Oxygenated Solutions of the Azo Dye Orange II. New J. Chem..

[16] Madhusudan P., Ran J., Zhang J., Yu J., Liu G. (2011). Novel Urea Assisted Hydrothermal Synthesis of Hierarchical BiVO_4_/Bi_2_O_2_CO_3_ Nanocomposites with Enhanced Visible-Light Photocatalytic Activity. Appl. Catal. B.

[17] Lachheb H., Puzenat E., Houas A., Ksibi M., Elaloui E., Guillard C., Herrmann J. (2002). Photocatalytic Degradation of various Types of Dyes (Alizarin S, Crocein Orange G, Methyl Red, Congo Red, Methylene Blue) in Water by UV-Irradiated Titania. Appl. Catal. B.

[18] Stylidi M., Kondarides D.I., Verykios X.E. (2003). Pathways of Solar Light-Induced Photocatalytic Degradation of Azo Dyes in Aqueous TiO_2_ Suspensions. Appl. Catal. B.

[19] Hu C., Yu J.C., Hao Z., Wong P.K. (2003). Photocatalytic Degradation of Triazine-Containing Azo Dyes in Aqueous TiO_2_ Suspensions. Appl. Catal. B.

[20] Zhang F., Zhao J., Shen T., Hidaka H., Pelizzetti E., Serpone N. (1998). TiO_2_-Assisted Photodegradation of Dye Pollutants II. Adsorption and Degradation Kinetics of Eosin in TiO_2_ Dispersions under Visible Light Irradiation. Appl. Catal. B.

[21] Tang W., Zhang Z., An H., Quintana M., Torres D. (1997). TiO_2_/UV Photodegradation of Azo Dyes in Aqueous Solutions. Environ. Technol..

[22] Tanaka K., Padermpole K., Hisanaga T. (2000). Photocatalytic Degradation of Commercial Azo Dyes. Water Res..

[23] Spadaro J.T., Isabelle L., Renganathan V. (1994). Hydroxyl Radical Mediated Degradation of Azo Dyes: Evidence for Benzene Generation. Environ. Sci. Technol..

[24] Augugliaro V., Baiocchi C., Bianco Prevot A., García-López E., Loddo V., Malato S., Marcí G., Palmisano L., Pazzi M., Pramauro E. (2002). Azo-Dyes Photocatalytic Degradation in Aqueous Suspension of TiO_2_ Under Solar Irradiation. Chemosphere.

[25] Konstantinou I.K., Albanis T.A. (2004). TiO_2_-Assisted Photocatalytic Degradation of Azo Dyes in Aqueous Solution: Kinetic and Mechanistic Investigations: A Review. Appl. Catal. B.

[26] Teh C.M., Mohamed A.R. (2011). Roles of Titanium Dioxide and Ion-Doped Titanium Dioxide on Photocatalytic Degradation of Organic Pollutants (Phenolic Compounds and Dyes) in Aqueous Solutions: A Review. J. Alloys Compd..

[27] Davis R.J., Gainer J.L., O’Neal G., Wu I. (1994). Photocatalytic Decolorization of Wastewater Dyes. Water Environ. Res..

[28] Houas A., Lachheb H., Ksibi M., Elaloui E., Guillard C., Herrmann J. (2001). Photocatalytic Degradation Pathway of Methylene Blue in Water. Appl. Catal. B.

[29] Paschoal F.M.M., Anderson M.A., Zanoni M.V.B. (2009). Simultaneous Removal of Chromium and Leather Dye from Simulated Tannery Effluent by Photoelectrochemistry. J. Hazard. Mater..

[30] Crini G. (2006). Non-Conventional Low-Cost Adsorbents for Dye Removal: A Review. Bioresour. Technol..

[31] Nohynek G.J., Fautz R., Benech-Kieffer F., Toutain H. (2004). Toxicity and Human Health Risk of Hair Dyes. Food Chem. Toxicol..

[32] Alves S.P., Brum D.M., Branco de Andrade É.C., Pereira Netto A.D. (2008). Determination of Synthetic Dyes in Selected Foodstuffs by High Performance Liquid Chromatography with UV-DAD Detection. Food Chem..

[33] Brown M.A., de Vito S.C. (1993). Predicting Azo Dye Toxicity. Crit. Rev. Environ. Sci. Technol..

[34] Tang W.Z., An H. (1995). UV/TiO_2_ Photocatalytic Oxidation of Commercial Dyes in Aqueous Solutions. Chemosphere.

[35] Galindo C., Jacques P., Kalt A. (2000). Photodegradation of the Aminoazobenzene Acid Orange 52 by Three Advanced Oxidation Processes: UV/H_2_O_2_, UV/TiO_2_ and VIS/TiO_2_: Comparative Mechanistic and Kinetic Investigations. J. Photochem. Photobiol. A.

[36] Saquib M., Muneer M. (2003). TiO_2_-Mediated Photocatalytic Degradation of a Triphenylmethane Dye (Gentian Violet), in Aqueous Suspensions. Dyes Pigments.

[37] Sakthivel S., Neppolian B., Shankar M., Arabindoo B., Palanichamy M., Murugesan V. (2003). Solar Photocatalytic Degradation of Azo Dye: Comparison of Photocatalytic Efficiency of ZnO and TiO_2_. Sol. Energy Mater. Sol. Cells.

[38] Guillard C., Lachheb H., Houas A., Ksibi M., Elaloui E., Herrmann J. (2003). Influence of Chemical Structure of Dyes, of pH and of Inorganic Salts on their Photocatalytic Degradation by TiO_2_ Comparison of the Efficiency of Powder and Supported TiO_2_. J. Photochem. Photobiol. A.

[39] So C., Cheng M.Y., Yu J., Wong P. (2002). Degradation of Azo Dye Procion Red MX-5B by Photocatalytic Oxidation. Chemosphere.

[40] Sauer T., Cesconeto Neto G., Jose H., Moreira R. (2002). Kinetics of Photocatalytic Degradation of Reactive Dyes in a TiO_2_ Slurry Reactor. J. Photochem. Photobiol. A.

[41] Daneshvar N., Salari D., Khataee A. (2004). Photocatalytic Degradation of Azo Dye Acid Red 14 in Water on ZnO as an Alternative Catalyst to TiO_2_. J. Photochem. Photobiol. A.

[42] Epling G.A., Lin C. (2002). Investigation of Retardation Effects on the Titanium Dioxide Photodegradation System. Chemosphere.

[43] Reutergådh L.B., Iangphasuk M. (1997). Photocatalytic Decolourization of Reactive Azo Dye: A Comparison between TiO_2_ and Us Photocatalysis. Chemosphere.

[44] Poulios I., Micropoulou E., Panou R., Kostopoulou E. (2003). Photooxidation of Eosin Y in the Presence of Semiconducting Oxides. Appl. Catal. B.

[45] Gomes de Moraes S., Sanches Freire R., Duran N. (2000). Degradation and Toxicity Reduction of Textile Effluent by Combined Photocatalytic and Ozonation Processes. Chemosphere.

[46] Gouvea C.A., Wypych F., Moraes S.G., Duran N., Nagata N., Peralta-Zamora P. (2000). Semiconductor-Assisted Photocatalytic Degradation of Reactive Dyes in Aqueous Solution. Chemosphere.

[47] Paz Y., Luo Z., Rabenberg L., Heller A. (1995). Photooxidative Self-Cleaning Transparent Titanium Dioxide Films on Glass. J. Mater. Res..

[48] Paz Y., Heller A. (1997). Photo-Oxidatively Self-Cleaning Transparent Titanium Dioxide Films on Soda Lime Glass: The Deleterious Effect of Sodium Contamination and its Prevention. J. Mater. Res..

[49] Lackhoff M., Prieto X., Nestle N., Dehn F., Niessner R. (2003). Photocatalytic Activity of Semiconductor-Modified Cement—Influence of Semiconductor Type and Cement Ageing. Appl. Catal. B.

[50] Benedix R., Dehn F., Quaas J., Orgass M. (2000). Application of Titanium Dioxide Photocatalysis to Create Self-Cleaning Building Materials. Lacer.

[51] Hashimoto K., Irie H., Fujishima A. (2005). TiO_2_ Photocatalysis: A Historical Overview and Future Prospects. Jpn. J. Appl. Phys..

[52] Murugan K., Rao T.N., Gandhi A.S., Murty B. (2010). Effect of Aggregation of Methylene Blue Dye on TiO_2_ Surface in Self-Cleaning Studies. Catal. Commun..

[53] Sakthivel S., Janczarek M., Kisch H. (2004). Visible Light Activity and Photoelectrochemical Properties of Nitrogen-Doped TiO_2_. J. Phys. Chem. B.

[54] Asahi R., Morikawa T., Ohwaki T., Aoki K., Taga Y. (2001). Visible-Light Photocatalysis in Nitrogen-Doped Titanium Oxides. Science.

[55] Sakthivel S., Kisch H. (2003). Daylight Photocatalysis by Carbon-modified Titanium Dioxide. Angew. Chem. Int. Ed..

[56] Ohno T., Mitsui T., Matsumura M. (2003). Photocatalytic Activity of S-Doped TiO2 Photocatalyst under Visible Light. Chem. Lett..

[57] Ho W. (2006). Low-Temperature Hydrothermal Synthesis of S-Doped TiO 2 with Visible Light Photocatalytic Activity. J. Solid State Chem..

[58] Li D., Haneda H., Labhsetwar N.K., Hishita S., Ohashi N. (2005). Visible-Light-Driven Photocatalysis on Fluorine-Doped TiO_2_ Powders by the Creation of Surface Oxygen Vacancies. Chem. Phys. Lett..

[59] Liu H., Gao L. (2004). (Sulfur, Nitrogen)-Codoped Rutile-Titanium Dioxide as a Visible-Light-Activated Photocatalyst. J. Am. Ceram Soc..

[60] Choi W., Termin A., Hoffmann M.R. (1994). The Role of Metal Ion Dopants in Quantum-Sized TiO_2_: Correlation between Photoreactivity and Charge Carrier Recombination Dynamics. J. Phys. Chem..

[61] Sant P.A., Kamat P.V. (2002). Interparticle Electron Transfer between Size-Quantized CdS and TiO_2_ Semiconductor Nanoclusters. Phys. Chem. Chem. Phys..

[62] Zou Z., Ye J., Arakawa H. (2001). Optical and Structural Properties of Solid Oxide Photocatalyst Bi_2_FeNbO_7_. J. Mater. Res..

[63] Luan J., Pan B., Paz Y., Li Y., Wu X., Zou Z. (2009). Structural, Photophysical and Photocatalytic Properties of New Bi_2_SbVO_7_ under Visible Light Irradiation. Phys. Chem. Chem. Phys..

[64] Nussbaum M., Shaham-Waldmann N., Paz Y. (2014). Synergistic Photocatalytic Effect in Fe, Nb-Doped BiOCl. J. Photochem. Photobiol. A.

[65] Tauc J., Grigorovici R., Vancu A. (1966). Optical Properties and Electronic Structure of Amorphous Germanium. Phys. Status Solidi (b).

[66] Yan X., Ohno T., Nishijima K., Abe R., Ohtani B. (2006). Is Methylene Blue an Appropriate Substrate for a Photocatalytic Activity Test? A Study with Visible-Light Responsive Titania. Chem. Phys. Lett..

[67] Ohtani B. (2010). Photocatalysis A to Z—What we Know and what we do Not Know in a Scientific Sense. J. Photochem. Photobiol. C.

[68] Bae S., Kim S., Lee S., Choi W. (2014). Dye Decolorization Test for the Activity Assessment of Visible Light Photocatalysts: Realities and Limitations. Catal. Today.

[69] Saison T., Chemin N., Chanéac C., Durupthy O., Ruaux V., Mariey L., Maugé F., Beaunier P., Jolivet J. (2011). Bi_2_O_3_, BiVO_4_, and Bi_2_WO_6_: Impact of Surface Properties on Photocatalytic Activity under Visible Light. J. Phys. Chem. C.

[70] Wang W., Huang F., Lin X. (2007). x-BiOI-(1-x)BiOCl as Efficient Visible-Light-Driven Photocatalysts. Scr. Mater..

[71] Zhang K., Liu C., Huang F., Zheng C., Wang W. (2006). Study of the Electronic Structure and Photocatalytic Activity of the BiOCl Photocatalyst. Appl. Catal. B.

[72] Jiang J., Zhao K., Xiao X., Zhang L. (2012). Synthesis and Facet-Dependent Photoreactivity of BiOCl Single-Crystalline Nanosheets. J. Am. Chem. Soc..

[73] Chen F., Liu H., Bagwasi S., Shen X., Zhang J. (2010). Photocatalytic Study of BiOCl for Degradation of Organic Pollutants under UV Irradiation. J. Photochem. Photobiol. A.

[74] Wang D., Gao G., Zhang Y., Zhou L., Xu A., Chen W. (2012). Nanosheet-Constructed Porous BiOCl with Dominant {001} Facets for Superior Photosensitized Degradation. Nanoscale.

[75] Dai K., Chen H., Peng T., Ke D., Yi H. (2007). Photocatalytic Degradation of Methyl Orange in Aqueous Suspension of Mesoporous Titania Nanoparticles. Chemosphere.

[76] Mills A., Wang J. (1999). Photobleaching of Methylene Blue Sensitised by TiO_2_: An Ambiguous System?. J. Photochem. Photobiol. A.

[77] De Tacconi N.R., Carmona J., Rajeshwar K. (1997). Reversibility of Photoelectrochromism at the TiO_2_/Methylene Blue Interface. J. Electrochem. Soc..

[78] Zhao Y., Swierk J.R., Megiatto J.D., Sherman B., Youngblood W.J., Qin D., Lentz D.M., Moore A.L., Moore T.A., Gust D. (2012). Improving the Efficiency of Water Splitting in Dye-Sensitized Solar Cells by using a Biomimetic Electron Transfer Mediator. Proc. Natl. Acad. Sci. USA.

[79] Zhao J., Wu T., Wu K., Oikawa K., Hidaka H., Serpone N. (1998). Photoassisted Degradation of Dye Pollutants. 3. Degradation of the Cationic Dye Rhodamine B in Aqueous Anionic Surfactant/TiO2 Dispersions under Visible Light Irradiation: Evidence for the Need of Substrate Adsorption on TiO2 Particles. Environ. Sci. Technol..

[80] Abe R., Hara K., Sayama K., Domen K., Arakawa H. (2000). Steady Hydrogen Evolution from Water on Eosin Y-Fixed TiO_2_ Photocatalyst using a Silane-Coupling Reagent under Visible Light Irradiation. J. Photochem. Photobiol. A.

[81] Pan L., Zou J., Zhang X., Wang L. (2011). Water-Mediated Promotion of Dye Sensitization of TiO_2_ under Visible Light. J. Am. Chem. Soc..

[82] Wang Q., Chen C., Zhao D., Ma W., Zhao J. (2008). Change of Adsorption Modes of Dyes on Fluorinated TiO_2_ and its Effect on Photocatalytic Degradation of Dyes under Visible Irradiation. Langmuir.

[83] Neppolian B., Choi H., Sakthivel S., Arabindoo B., Murugesan V. (2002). Solar Light Induced and TiO_2_ Assisted Degradation of Textile Dye Reactive Blue 4. Chemosphere.

